# Clinical Utility of Robot-Assisted Gait Training in Patients with Spinal Cord Injury Caused by Electrical Burns: A Case Report

**DOI:** 10.3390/jcm12237220

**Published:** 2023-11-21

**Authors:** Seung-Yeol Lee, Cheong-Hoon Seo, Yoon-Soo Cho, So-Young Joo

**Affiliations:** 1 Department of Physical Medicine and Rehabilitation, College of Medicine, Soonchunhyang University Hospital, Bucheon 14584, Republic of Korea; shouletz@gmail.com; 2 Department of Rehabilitation Medicine, College of Medicine, Hangang Sacred Heart Hospital, Hallym University, Seoul 07247, Republic of Korea; chseomd@gmail.com (C.-H.S.); hamays@hanmail.net (Y.-S.C.)

**Keywords:** robot-assisted gait training, spinal cord injury, burns, electrical trauma

## Abstract

Robot-assisted gait training (RAGT) has been proven effective in improving gait function in not only patients with central nervous system damage, but also in patients who have undergone musculoskeletal surgery. Nevertheless, evidence supporting the efficacy of such training in burn patients remains insufficient. This report aimed to evaluate the effect of RAGT in burn patients with spinal cord injuries (SCI) caused by electrical trauma. We reported a case of two patients. The total duration of each session was about 1 h 30 min. This included 10 min to put on the exoskeleton, 30 min of robot-assisted training using SUBAR^®^, 10 min to remove the exoskeleton, 10 min to observe whether complications such as skin abrasion, ulcer, or pain occur in the scar area after RAGT, and 30 min of conventional physiotherapy, at a rate of 5 days a week for 12 weeks. All measurements were assessed before training (0 week) and after training (12 weeks). The American Spinal Cord Injury Association (ASIA) lower extremity motor score (LEMS), passive range of motions (ROMs) of different joints (hip, knee, and ankle), ambulatory motor index (AMI), functional ambulation categories (FAC), and 6 min walking (6 MWT) distances were evaluated to measure the degree of gait function through training. In both patients, manual muscle test measurement and joint ROM in the lower extremities improved after 12 weeks training. The first patient scored 0 in the FAC before training. After 12 weeks of training, he could walk independently indoors, improving to an FAC score of 4. He also reached 92.16 m in the 6 MWT. LEMS improved from 22 before training to 30 after training, and AMI score improved from 12 before training to 16 after training. In the second patient, an independent walking function was not acquired. LEMS improved from 10 before training to 26 after training. AMI scores were the same at 10 points before and after training. The results suggested the possibility of achieving clinical effects in terms of improving lower extremity muscle strength, joint ROMs, and gait performance in patients with SCI caused by electrical trauma.

## 1. Introduction

The incidence of spinal cord injury (SCI) following high-voltage electrical trauma ranges between 2 and 5%. SCI can occur early or late after trauma. Early SCI occurs in patients within a few hours of the injury and they recover within a few days [1]. Delayed SCI occurs a few days to a few months after electrical trauma and becomes permanent as incomplete SCI [1,2]. Tissue mechanisms caused by electricity include trauma, ischemic injury, heating effect, and electroporation [3]. The heating effect causes early SCI, and cell membrane rupture due to electroporation causes delayed SCI. Ischemic injury caused by endothelial injury or thrombosis is also a major cause of delayed SCI [4,5]. There is a lot of damage to the anterior spinal cord, where many blood vessels are distributed, and motor tracks passing through this area are damaged, causing motor function deterioration [4]. However, there is no specific imaging test used to diagnose SCI caused by electrical trauma, and imaging tests are often judged to be normal [6]. When symptoms of SCI occur, imaging test results are usually normal. Thus, the diagnosis of neurological injury is based on progress and clinical findings. Early recognition of SCI could be important to initiating intensive rehabilitation as early as possible. Patients with SCI have varying degrees of muscle weakness, spasticity, and impaired balance. The most challenging complication of SCI is walking impairment [7]. Additionally, hypertrophic scarring caused by electrical burns causes joint contracture and sensorimotor disturbances, which reduces gait function [8].

Conventional physical therapy for a patient with SCI focuses on muscle strengthening, stretching, and manually assisted gait training. Manually assisted gait training helps to strengthen lower extremity muscles and improve walking ability. As in normal walking, exercises to move the lower extremities are required to activate the locomotor centers in the spinal cord. However, in reality, manually assisted gait training by therapists requires significant effort from the therapist, and long-term training is not possible. Patients who cannot walk independently need the help of a mechanical system to improve their walking function.

Robot-assisted gait training (RAGT) positively affects lower extremity muscle strength, gait speed, walking performance, and gait pattern in patients with central nervous system damage such as SCI or stroke [9,10,11,12,13]. The therapeutic area of robot therapy is expanding to improve the function of musculoskeletal patients who have undergone arthroplasty or burn skin grafting [14,15,16].

However, there is still no research on the application of robot training to improve gait function in patients with combined SCI and hypertrophic scars caused by electrical trauma. It was assumed that RAGT would have a positive effect in terms of improving gait function in patients with SCI caused by high-voltage electricity. The objective of this study was to demonstrate that the RAGT for 12 weeks with conventional physiotherapy can lead to clinically meaningful improvement in patients with SCI caused by electrical trauma.

## 2. Case Reports

This study was approved by the Ethics Committee of Hangang Sacred Heart Hospital (HG2021-001). This study was registered at ClinicalTrials.gov (identifier: NCT05883917). The patients provided written informed consent. The inclusion criteria were (1) age between 18 and 75 years old; (2) high-voltage electrical injury at more than 1000 volts; (3) motor incomplete spinal cord injury or upper motor neuron injuries with an NLI of T10 or higher; (4) severity (American Spinal Cord Injury Association Impairment Scale [ASIA]) of B or C or D; (5) no current issues with or history of other neurological conditions; and (6) involvement in standing program or ability to tolerate at least 30 min upright without signs or symptoms of orthostatic hypotension. The exclusion criteria were (1) ASIA A; (2) lower motor neuron injuries, as shown by absent reflexes during bilateral quadriceps and Achilles tendon taps; (3) previous history of spinal cord disease; (4) abnormal lesions in radiologic evaluations taken after development of extremity weakness; (5) orthostatic hypotension that makes training impossible during 30 min; (6) recent lower extremity fracture; (7) fixed contractures that make it impossible to apply the robot; and (8) skin lesions at the contact areas with the exoskeleton. We recruited 2 patients from the Department of Rehabilitation Medicine at Hangang Sacred Heart Hospital in Korea to participate in this study between January 2021 to December 2021.

### 2.1. Therapeutic Intervention

Before robot training, it was recommended to apply scar lubrication and wear a compression garment to prevent complications in the scar area. Gait training was conducted using SUBAR^®^ (Cretem, Anyang-si, Republic of Korea), which is a wearable robot with a footplate that assists in improving patient gait [8,14,17,18]. The SUBAR^®^ is an exoskeleton robot that can adjust step length, gait speed, and knee flexion angle. The SUBAR^®^ allows voluntary trunk muscles and reciprocal movements of the upper limbs when gait training and allows passive movements of the lower limb according to the adjusted parameters. The patient’s thigh length and lower leg length were measured before training so that the SUBAR^®^ could be adjusted to the patient’s size in order to ensure accurate training. One physiotherapist, who has experience in SUBAR^®^, assisted the patients with wearing exoskeleton robot and setting the training intensity. Periodic movement of the lower extremities during training was simulated at a tolerable and comfortable gait speed, adjusted to the gait speed of each patient.

Conventional physiotherapy comprised 30 min of traditional gait training (extremity mobilization, strengthening and stretching exercises for the lower limbs, and gait re-education when participants can stand or walk). The total duration of each session was about 1 h 30 min, which included 10 min to put on the exoskeleton, 30 min of robot-assisted training using SUBAR^®^, 10 min to remove the exoskeleton, 10 min to observe whether complications occur in the scar area after RAGT, and 30 min of conventional physiotherapy, at a rate of 5 days a week for 12 weeks.

### 2.2. Outcome Measure

All measurements were assessed before training (0 week) and after training (12 weeks). Using the standards for Neurological Classification of Spinal Cord Injury (ISNCSCI) [19], 28 dermatomes on the bilateral side of SCI patients were evaluated for sensory sensitivity to light touch and pin prick testing. The sensitivities were determined on the three-point scale of absent (0), impaired (1), or normal (2). Each ASIA sensory score for light tough and pin prick is a summation, falling within a 0–112 range of the scores for all 28 dermatomes. The manual muscles tests (MMTs) of ten key muscles were evaluated and graded on a six-point scale between 0 (complete paralysis) and 5 (normal active movements, full range of motion against full resistance). These scores were aggregated to generate an ASIA motor score within the 0–100 range. The ASIA lower extremity motor subscale score (LEMS; range 0–50) was used to evaluate the motor function. LEMS is the sum of bilateral lower extremity key muscle power, ranging from total paralysis (0) to normal active movement with a full range of motion against gravity and maximum resistance (5), with a total possible score of 50 [20]. The passive range of motions (ROMs) of different joints (hip, knee, and ankle) were measured using a goniometer [21]. The ambulatory motor index (AMI; range 0–30), which predicts ambulatory capability, was measured by evaluating muscles of hip flexion, hip abduction, hip extension, knee extension, and knee flexion on both sides [22]. Functional ambulation categories (FAC) were evaluated based on a 6-point scale, ranging from unable to walk (0), dependency in gait (1 or 2), gait on even and level surfaces without manual contact with another person except for safety (3), and independent gait over 15 m irrespective of aids used (4 or 5). Walking distances of 6 min (6 MWT) were in accordance with the standardized guidelines, and the walking course was 20 m. The patients were instructed to walk as far as possible in 6 min [23].

### 2.3. Case Presentation

#### 2.3.1. Patient 1

The first patient (male, 48 years old) was admitted to the hospital after suffering an electrical trauma (22,900 V) without loss of consciousness. On admission, the patient was alert and oriented. No neurological deficits were present. A 28% burn of the total body surface area (TBSA) was identified on the frank, bilateral thighs, and bilateral legs. After 14 days of hospitalization, signs of SCI, such as exacerbated knee reflex, Babinski’s sign, abnormal sensation, and loss of leg strength, were observed. Owing to a developing lower extremity weakness, contrast-enhanced CT and MRI using T1- and T2-weighted sequences of the spine were performed, the results of which were normal. The patient had difficulty walking because of weakness in the lower extremities and complained of paresthesia. A split-thickness skin graft (STSG) was performed on the left flank and both thighs a month after the injury. One week after the STSG, balance training and lower extremity muscle strengthening exercises were started in bed by a physical therapist. After the completion of epithelialization of the burn scar, the patient was admitted to the Department of Rehabilitation Medicine. He started a RAGT on the 56th day of the injury for lower extremity weakness and gait dysfunction. Before training, the patient’s physical condition showed a neurological level of injury (NLI) of T 10, an ASIA impairment scale grade C (Table 1), an LEMS of 22 points, and an AMI of 12 points, and he was able to sit independently.

However, he was unable to stand or walk independently. In an MMT, the flexor (right/left), extensor (right/left), abductor (right/left), and adductor (right/left) of the hip joint improved from 2/2, 2/2, 2/2, and 1/1 before training to 3/3, 2/2, 3/3, and 2/2 after training. No differences were observed in knee flexor (right/left) and knee extensor (right/left), with scores of 2/2 and 3/3 before training and 2/2 and 3/3 after training. The ankle dorsiflexor (right/left) and ankle plantarflexor (right/left) improved from 2/2 and 2/2 before training to 3/3 and 3/3 after training. The long toe extensor (right/left) improved from 2/2 before training to 3/3 after training (Table 2). In passive ROM evaluation, hip flexion (right/left) ROMs improved from 60°/60° before training to 100°/100° after training. And there were no differences in the hip extension (right/left) ROMs, moving from 5°/5° before training to 5°/5° after training. The knee flexion (right/left) ROMs improved from 100°/105° before training to 105°/105° after training. There was no difference in the knee extension (right/left) ROMs, moving from 0°/0° before training to 0°/0° after training. The ankle dorsiflexion (right/left) ROMs improved from 5°/5° before training to 10°/10° after training. There was no difference in the ankle plantarflexion (right/left) ROMs, moving from 40°/40° before training to 40°/40° after training (Table 2).

Patient 1, who was unable to walk before training, was able to walk indoors after 12 weeks training. After 12 weeks of RAGT, he could walk 92.16 m in the 6 MWT. LEMS improved from 22 before training to 30 after training, and AMI scores improved from 12 before training to 16 after training (Table 3).

#### 2.3.2. Patient 2

**Figure 1:**
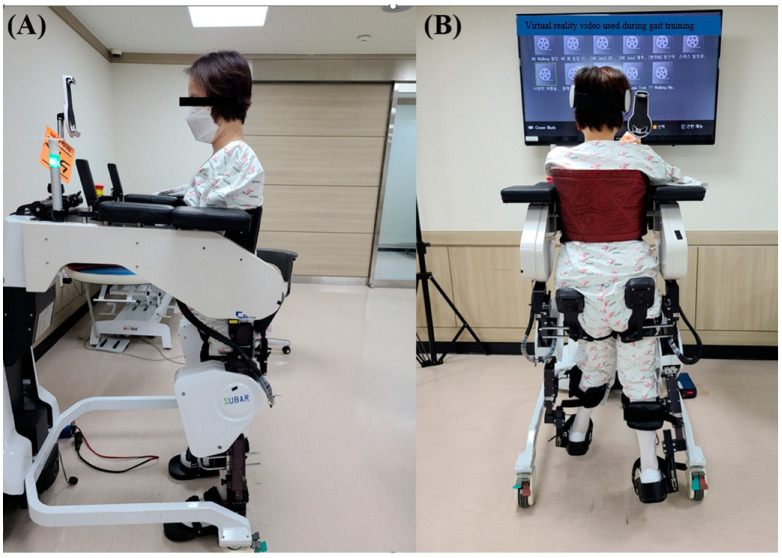
Robot training in Case 2: lateral (**A**) and posterior (**B**) views.

The second patient (female, 45 years old) was admitted to the hospital after an electrical trauma (22,900 V). A 29% burn of TBSA was identified on the abdomen, right inguinal, bilateral arms, bilateral forearms, and bilateral hands. On the day of hospitalization, escharectomy and fasciotomy were performed on the upper extremity scars, and 12 days after injury, bilateral transhumeral amputation was performed owing to the inflammatory reaction and decay of the upper limb scars on both sides. Motor weakness and paresthesia of both lower extremities occurred 5 weeks after the injury. She was referred to the Department of Rehabilitation Medicine. Signs of SCI appeared, such as bilaterally exacerbated knee reflexes, bilateral Babinski’s sign, and decreased sensation. Contrast-enhanced CT and MRI using T1- and T2-weighted sequences of the spine at this time revealed normal results. After the epithelialization of the burn scar was completed, she was admitted to the Department of Rehabilitation Medicine. She could not sit independently due to muscle weakness in her trunk; therefore, sitting training, and trunk strengthening exercises were performed when she was first admitted. Notably, 110 days after the injury, the trunk muscles became strong enough to allow her to sit independently for 5 min, and RAGT was started. RAGT was performed for 30 min using a trunk support harness because of trunk muscle weakness (Figure 1).

The patient’s physical condition showed an NLI C4, ASIA impairment scale grade C (Table 1), LEMS of 10 points, and AMI of 10 points. Independent sitting was possible for 5 min; however, independent standing and walking were impossible before training (Table 3). In an MMT, the flexor (right/left), extensor (right/left), abductor (right/left), and adductor (right/left) of the hip joint improved from 1/1, 1/1, 1/1, and 1/1 before training to 2/2, 2/2, 2/2, and 2/2 after training. The knee flexor (right/left) and knee extensor (right/left) improved from 1/1 and 1/1 before training to 2/2 and 2/2 after training. The ankle dorsiflexor (right/left) and ankle plantarflexor (right/left) improved from 1/1 and 1/1 before training to 3/3 and 3/3 after training. The long toe extensor (right/left) improved from 1/1 before training to 3/3 after training (Table 2). In passive ROM evaluation, hip flexion (right/left) ROMs improved from 90°/90° before training to 95°/95° after training. And there were no differences in the hip extension (right/left) ROMs from 5°/5° before training to 5°/5° after training. The knee flexion (right/left) ROMs improved from 120°/120° before training to 130°/130° after training. There was no difference in the knee extension (right/left) ROMs from 0°/0° before training to 0°/0° after training. The ankle plantarflexion (right/left) ROMs improved from 35°/35° before training to 45°/45° after training. There was no difference in the ankle dorsiflexion (right/left) ROMs, with 10°/10° before training and 10°/10° after training (Table 2). She could not walk independently. LEMS improved from 10 before training to 26 after training. AMI scores were the same at 10 points before and after training (Table 3).

The patients could physically tolerate the RAGT, all sessions were completed, and no adverse events occurred.

## 3. Discussion

After 12 weeks of RAGT and conventional physiotherapy in patients with delayed SCI due to high-voltage electrical burns, the first patient’s lower limb strength and joint ROMs improved, and the walking function recovered to the point where indoor walking was possible. In the second patient, walking function was not recovered, but lower limb strength and ROMs were confirmed to be improved.

Joint contractures and movement restrictions in the lower extremities interfere with normal walking and act as factors that decrease gait speed and reduce the step length [14]. We used 6 MWT to quantitatively evaluate gait ability in patients with incomplete SCI [23]. LEMS is the most important factor in predicting gait ability with incomplete SCI. A previous study indicated that the prospects for gait improvement are poor if LEMS is less than 20, but that the prognosis is good if LEMS is more than 30 [24]. Efforts are made to improve these parameters that affect gait function [25].

The clinical effect of applying robot therapy to burn patients is described as the influence of task-specific training on walking performance or hand function [8,14]. It was confirmed that joint ROM, muscle strength, and gait speed improved when robot training was applied to patients with decreased lower extremity function due to thermal trauma [8,14]. The reduced arthrogenic muscle inhibition during robot treatment for patients is also considered a mechanism for improving performance. Sensory deficits, muscle weakness, abnormal muscle activities, proprioception impairments, and soft tissue tightness in patients with SCI can impair walking ability [26,27]. RAGT can increase the repetition of training while maintaining a physiological gait pattern [28]. The mechanism underlying gait function restoration after SCI involves enhancing the sensory and proprioception input to the spinal cord [29] and activating the central gait pattern generator [22]. Plastic changes can be induced at the spinal cord level and in the sensory–motor cortex. This means that robot training changes the gait center of the spine and supraspinal area [10]. RAGT in the acute phase improves muscle strength and gait speed through muscle activation patterns and gait pattern relearning [30]. Improvement in cardiopulmonary capacity via robot training improves gait speed and endurance. Trunk control during robot training is an important factor that affects gait [9]. One case report demonstrated how robot gait training engages trunk muscles and can elicit training effects on balance control in patients with SCI [31]. RAGT is a promising technique for restoring functional walking and improving the gait ability of SCI patients [9,28].

This study was performed on the results of RAGT and conventional physiotherapy in only two patients with delayed SCI due to electrical trauma. Although the results were confirmed, there were limitations in that the clinical effect could not be confirmed when applied to all patients with SCI caused by electrical trauma. In addition, the improvement in the patient’s lower extremity function could not be entirely explained by robot training as RAGT and conventional physiotherapy were performed simultaneously. Further research should seek to confirm that improvement in gait parameters after robot training exceeds that achieved after conventional training to validate the effectiveness of RAGT. Studies employing multiple patients and control groups are necessary to demonstrate the effectiveness and treatment mechanisms of RAGT in patients with SCI after an electrical burn.

## 4. Conclusions

This study is the first to report the feasibility, safety, and efficacy of RAGT for patients with SCI after electrical burns. The robot training was easily performed in patients with SCI caused by electrical burns without any undesirable side effects. The results suggested the possibility of achieving clinical effects in terms of improving lower extremity muscle strength, joint ROMs, and gait performance in patients with SCI caused by electrical trauma.

## Figures and Tables

**Table 1:** Patients’ demographic and clinical data.

	Case 1	Case 2
Age (years)	48	45
Gender	Male	Female
Injury level	T10	C4
ASIA grade	C	C
Post-injury (days)	56	110
TBSA	28	29

TBSA, total body surface area; ASIA, American Spinal Cord Injury Association impairment scale.

**Table 2:** Manual muscle test (MMT) measurements and range of motion (ROM) before and after training.

	Case 1		Case 2	
	Before Training	After Training	Before Training	After Training
**Muscle muscles test (MMT)**				
Hip				
Flexor, right	2	3	1	2
Flexor, left	2	3	1	2
Extensor, right	2	2	1	2
Extensor, left	2	2	1	2
Abductor, right	2	3	1	2
Abductor, left	2	3	1	2
Adductor, right	1	2	1	2
Adductor, left	1	2	1	2
Knee				
Flexor, right	2	2	1	2
Flexor, left	2	2	1	2
Extensor, right	3	3	1	2
Extensor, left	3	3	1	2
Ankle				
Dorsiflexor, right	2	3	1	3
Dorsiflexor, left	2	3	1	3
Plantar flexor, right	2	3	1	3
Plantar flexor, left	2	3	1	3
Toe				
Long toe extensor, right	2	3	1	3
Long toe extensor, left	2	3	1	3
**Range of motion (degree)**				
Hip				
Flexion, right	60	100	90	95
Flexion, left	60	100	90	95
Extension, right	5	5	5	5
Extension, left	5	5	5	5
Knee				
Flexion, right	100	105	120	130
Flexion, left	105	105	120	130
Extension, right	0	0	0	0
Extension, left	0	0	0	0
Ankle				
Dorsiflexion, right	5	10	10	10
Dorsiflexion, left	5	10	10	10
Plantarflexion, right	40	40	35	45
Plantarflexion, left	40	40	35	45

**Table 3:** Walking capacities before and after training.

	Case 1		Case 2	
	Before Training	After Training	Before Training	After Training
6 MWT	0	92.16	0	0
FAC	0	4	0	0
LEMS	22	30	10	26
AMI	12	16	10	10

FAC, functional ambulation categories; 6 MWT, 6 min walking test; LEMS, lower extremity motor subscale; AMI, ambulatory motor index.
